# Characterization of Spontaneous Immune Responses against Long Peptides Derived from Bcl-X(L) in Cancer Patients Using Elispot

**DOI:** 10.3390/cells1020051

**Published:** 2012-04-26

**Authors:** Stine Kiaer Larsen, Morten Hansen, Inge Marie Svane, Per Thor Straten, Mads Hald Andersen

**Affiliations:** Center for Cancer Immune Therapy (CCIT), Department of Hematology, Copenhagen University Hospital, Herlev, Herlev Ringvej 75, DK-2730 Herlev, Denmark; Email: stine.kiaer.larsen@regionh.dk (S.K.L.); Morten.Hansen.01@regionh.dk (M.H.); inge.marie.svane@regionh.dk (I.M.S.); per.thor.straten@regionh.dk (P.T.S.)

**Keywords:** ELISPOT, Bcl-X(L), long peptides, antigen, T cells

## Abstract

In recent years we and others have used the ELISPOT assay successfully to identify novel tumor antigens by the characterization of spontaneous HLA class I restricted immune responses against a number of minimal 9–10 amino acid long peptide epitopes. In the present study, we examined the capability of using longer peptides when scrutinizing Peripheral Blood Mononuclear Cells (PMBC) from melanoma patients for spontaneous immunity by means of ELISPOT IFN-γ secretion assay. To this end, we examined PBMC for the presence of specific T-cell responses against long peptides derived from the tumor associated antigen BCL-X(L). The protein product of the larger BCL-X(L) differs from Bcl-X(S) protein by an inserted region (amino acids 126–188). Thus, we scrutinized eight long peptides covering this inserted region for spontaneous immunity. The peptides were overlapping and consisted of 20–23 amino acids. PBMC were pre-stimulated with peptide-pulsed autologous dendritic cells (DC) and subjected to the IFN-γ ELISPOT assay. Four of the BCL-X(L) derived peptides elicited very frequent responses in several patients. Additionally, in all patients responses against more than one of the peptides could be detected. In conclusion several long BCL-X(L) derived peptide epitopes exist, which may be used in anti-cancer immunity. Furthermore, the ELISPOT assay offers an attractive and sensitive method for the characterization of spontaneous immune reactivity against long peptides.

## 1. Introduction

For many years the measurements of the levels of cellular immune responses, e.g., those mediated by T cells, depended largely on *in vitro* culture and subsequent measurement of specific functions like cytotoxicity, proliferation or bulk cytokine production. Importantly, new approaches to monitor and analyze anti-tumor immune responses, requiring minimal *in vitro* manipulations have opened new avenues to characterize spontaneous as well as treatment-induced T-cell responses [1]. To this end, technical advantages allow the detection of low frequencies of precursor CD8^+^ T cells with high sensitivity. Among the different methods available for monitoring of CD8^+^ T cells responses due to its high throughput, sensitivity and robustness the ELISPOT assay represents the method of choice in many laboratories. The ELISPOT assay is based on the detection of antigen-induced release of cytokines—most often IFN-γ—by single T cells upon triggering of its TCR [2]. Reactivity of a single T cell can be detected and quantified via binding of the respective cytokine on special nitro-cellulose filter plates. For this purpose, cytokine specific antibodies are coated to the nitro-cellulose to capture secreted cytokines. Target cells, e.g., peptide-pulsed TAP-deficient T2 cells, are incubated together with the cell preparation, which is analyzed whether it contains antigen reactive T-cells. When a T cell recognizes the peptide epitope examined, the T cell releases cytokines that is detected by a colorimetric reaction using an enzyme conjugated to a second cytokine specific antibody. The reaction product is visible as a spot. Ideally, each spot represents the cytokines secreted by a single activated cell. In cases when responses are suspected to be weak an *in vitro* stimulation can be used to enhance sensitivity of the assay. 

The ELISPOT have proven to be the central assays in studies focusing on identification of novel tumor antigens by the characterization of spontaneous class I HLA-restricted CD8 T-cell responses in PMBC from cancer patients. Thus, this approach has previously proved to be highly effective for identifying tumor specific cytotoxic T-lymphocytes (CTL) in cancer patients [3,4,5]. For these assays, minimal peptide epitopes have been selected on the basis of HLA-binding motifs using the main HLA-specific anchor residues [6] or different predictive computer algorithms, e.g., the one developed by Rammensee *et al.* available at www.syfpeithi.de. Longer peptides than minimal 9–10 amino acid may contain not only CD8 T cell epitopes but in addition CD4 T helper epitopes. Furthermore, if used in a clinical setting, e.g., for anti-cancer vaccinations, longer peptides may specifically target professional antigen presenting cells, which are capable of the up taking and processing into HLA of larger peptide antigens. 

Using the ELISPOT it has previously been demonstrated that breast cancer patients, melanoma patients and pancreatic cancer patients host spontaneous HLA class I-restricted CD8 T-cell responses specifically against 9–10 amino acid long Bcl-X(L)-derived peptides [7]. In the present study we examined the capability of using longer peptides when scrutinizing PMBC from melanoma patients for spontaneous immunity by means of ELISPOT IFN-γ secretion assay.

## 2. Materials and Methods

### 2.1. Donors

Peripheral Blood Mononuclear Cells (PBMC) was collected from melanoma patients. The PBMC were obtained prior to entering into a clinical trial, which were concurrently approved by the Danish Medicines Agency and registered at www.clinicaltrials.gov (NCT00978913). Written informed consent from the donors was obtained before study entry. All patients had histological verified metastatic disease (stage IV TNM classification) at inclusion. Blood samples from cancer patients were drawn a minimum of four weeks after termination of any kind of anti-cancer therapy. PBMC were isolated using lymphoprep separation, HLA-A2 typed (Department of Clinical Immunology, University Hospital, Copenhagen, Denmark) and frozen in FCS with 10% DMSO. The protocols were approved by the Scientific Ethics Committee for The Capital Region of Denmark and conducted in accordance with the provisions of the Declaration of Helsinki.

### 2.2. Peptides

The *bcl-x* gene is transcribed into two mRNAs through alternative splicing. The anti-apoptotic protein Bcl-X(L) is produced from the long isoform, while pro-apoptotic Bcl-X(S) is derived from the short isoform mRNA [8]. The protein product of the larger Bcl-X(L) differs from Bcl-X(S) protein by an inserted region (amino acids 126–188). Eight synthetic peptides covering this inserted region (including nine amino acids at each end) were synthesized (TAG Copenhagen, Copenhagen, Denmark): Bcl-X1(TAYQSFEQVVNELFRDGVNW), Bcl-X2 (VVNELFRDGVNWGRIVAFFS), Bcl-X3 (GVN WGRIVAFFSFGGALCVE), Bcl-X4 (AFFSFGGALCVESVDKEMQV), Bcl-X5 (LCVESVDKEMQ VLVSRIAAW), Bcl-X6 (EMQVLVSRIAAWMATYLNDH), Bcl-X7 (IAAWMATYLNDHLEPWI QEN) as well as Bcl-X8 (LNDHLEPWIQENGGWDTFVELYG). 

### 2.3. Generation of Dendritic Cells

Dendritic cells were generated from PBMC by plate adherence and five days of incubation with 1,000 U/mL GM-CSF and 250 U/mL of IL-4 in X-VIVO with 5% AB-serum. Maturation was induced for two additional days with a standard cytokine cocktail containing 1,000 U/mL TNF-α, 1,000 U/mL IL-1β, 1,000 U/ml IL-6 and 1 ug/mL PGE2. 

### 2.4. Phenotype of Mature DCs by Flow Cytometry

DCs were pre-blocked with human immunoglobulin and stained with antibodies and matched isotype controls against CCR7 (R&D Systems), CD83, CD80, HLA-DR, CD86, and CD40 (BD Bioscience) and dead cells were excluded by staining with 7-amino-actinomycin D (from Sigma-Aldrich). DCs were analyzed using a BD FACSCanto II flow cytometer and data were processed using FlowJo (Treestar).

### 2.5. ELISPOT Assay

The ELISPOT assay was used to quantify peptide-specific IFN-γ releasing effector cells as described [3]. PBMC were stimulated once *in vitro* with autologous dendritic cells (DC) pulsed with peptide prior to analysis [9] to extend the sensitivity of the assay. After seven days in culture with 25 µg/mL peptide and 40 U/mL IL-2 (PeproTech, London, UK), cells were counted and analyzed in ELISPOT. Briefly, nitrocellulose bottomed 96-well plates (MultiScreen MAIP N45; Millipore) were coated overnight with IFN-γ capture mAb (Mabtech, Nacka Strand, Sweden). The wells were washed, blocked by X-vivo medium. PBMC at different cell concentrations with 10^4^ autologous DC with or without 5 µg/mL of the appropriate peptide were added in duplicates. The plates were incubated overnight. The following day, medium was discarded and the wells were washed prior to addition of appropriate biotinylated secondary mAb (Mabtech). The plates were incubated at room temperature (RT) for 2 hours, washed, and Avidin-enzyme conjugate (AP-Avidin; Calbiochem/Invitrogen Life Technologies) was added to each well. Plates were incubated at RT for 1 hour and the enzyme substrate Streptavidin-ALP (Mabtech) was added to each well and incubated at RT for 5–10 min. Upon the emergence of dark purple spots, the reaction was terminated by washing with tap water. The spots were counted using the ImmunoSpot Series 2.0 Analyzer (CTL Analyzers). 

## 3. Results

### 3.1. Dendritic Cells

**Figure 1:**
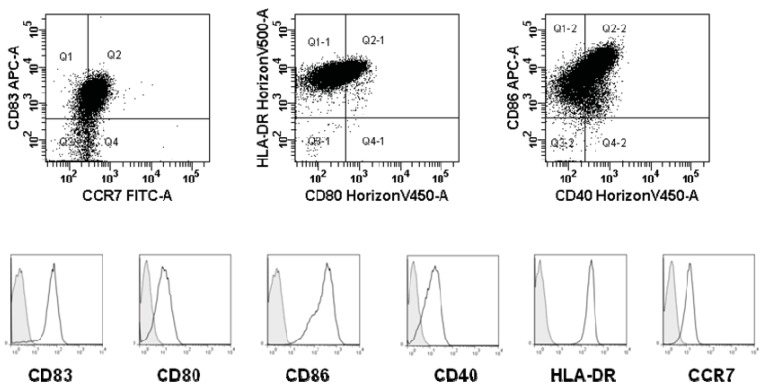
An example of phenotype (expression of CCR7, CD83, CD80, HLA-DR, CD86, and CD40) of standard matured DC. Overlay histograms of maturation markers (black curve) and matched isotype controls (tinted, dotted curve) is shown.

Phenotypic expression of maturation markers (CD83, CD80, CD86, CD40, HLA-DR, CCR7) were confirmed by flow cytometry on harvested DCs. A typical example of a mature standard DC is given in Figure 1. However, the DC did not seem to produce any IL-12 (data not shown).

### 3.2. ELISPOT Responses against Long Bcl-X(L) Derived Peptides

The protein product of the larger BCL-X_L_ differs from Bcl-X_S_ protein by an inserted region (amino acids 126–188). We selected and synthesized eight 20–23 amino acid overlapping peptides spanning this inserted region. Using the ELISPOT IFN-γ secretion assay, we examined peripheral blood T cells from eight melanoma patients for the presence of specific T-cell responses against these Bcl-X(L) derived peptides. First, PBMC were stimulated once *in vitro* before examination by ELISPOT.

**Figure 2:**
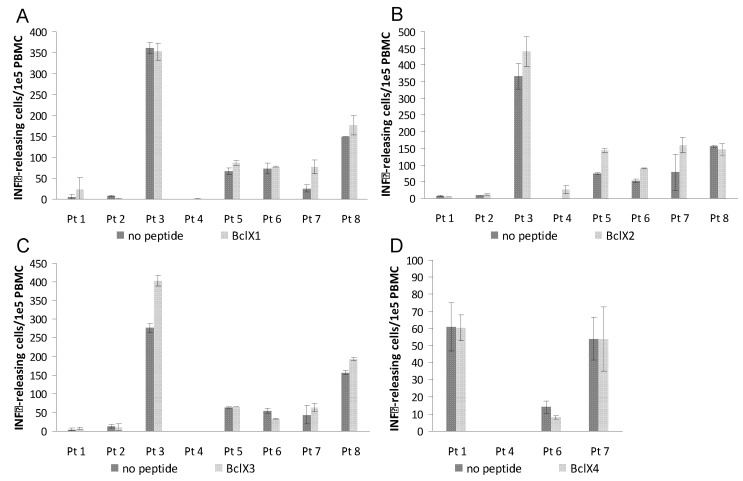
T-cell responses against Bcl-X(L) as measured by IFN-γ ELISPOT. PBMC from eight melanoma patients were analyzed. The peptides Bcl-X1(TAYQSFEQVVNELFR DGVNW) **(A)**, Bcl-X2 (VVNELFRDGVNWGRIVAFFS) **(B)**, Bcl-X3 (GVNWGRIVAFF SFGGALCVE)** (C)**, and Bcl-X4 (AFFSFGGALCVESVDKEMQV) **(D)** were examined. T-lymphocytes were stimulated once with peptide before being plated at 10^5^ cells per well in duplicates either with or without peptide. The number of IFN-γ without (*dark grey*) or with (*light gray*) added peptide was calculated for each patient using the ImmunoSpot® Series 2.0 Analyzer (CTL Analyzers, LLC, Cleveland, USA).

**Figure 3:**
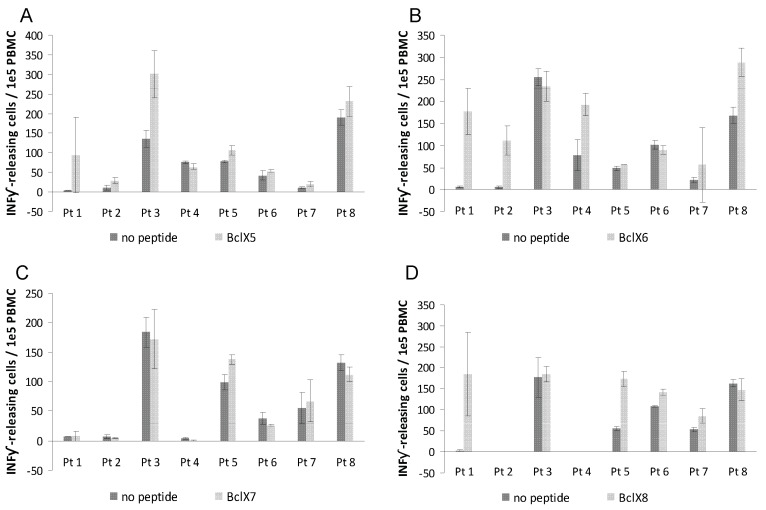
T-cell responses against Bcl-X(L) as measured by IFN-γ ELISPOT. PBMC from eight melanoma patients were analyzed. The peptides Bcl-X5 (LCVESVDKEMQVLVSRI AAW) **(A)**, Bcl-X6 (EMQVLVSRIAAWMATYLNDH)** (B)**, Bcl-X7 (IAAWMATYLND HLEPWIQEN) **(C)**, and Bcl-X8 (LNDHLEPWIQENGGWDTFVELYG) **(D)** were examined. T-lymphocytes were stimulated once with peptide before being plated at 10^5^ cells per well in duplicates either without or with peptide. The number of IFN-γ without (*dark grey*) or with (*light gray*) added peptide was calculated for each patient using the ImmunoSpot® Series 2.0 Analyzer (CTL Analyzers, LLC, Cleveland, USA).

After 7 days of culture PBMC were screened in ELISPOT assays for release of IFN-γ upon peptide recognition. Autologous DC were used as antigen presenting cells (APC) in the ELISPOT assay. Responses were detected against four peptides BclX2, BclX5, BclX6 and BclX8 (Figure 2 and Figure 3) Hence, four of examined patients hosted an immune response against BclX6, whereas we detected a response against BclX8 in two patients. Similary we detected responses against BclX5 in two patients. Finally we detected some weak responses against BclX2. In one patient we could detect a response against BclX3, however in this patient there was in addition high background response. Overall, the responses against the Bcl-X(L)-derived peptides were frequent responses, since responses could be detected in all patients (Figure 2 and Figure 3). 

**Figure 4:**
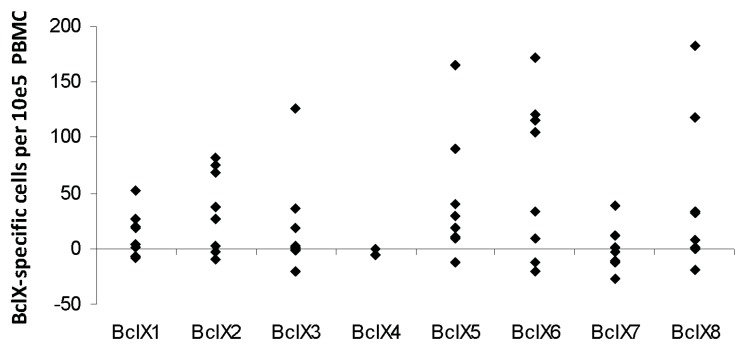
Screening of IFN-γ producing T-cell responses against BclX(L)-derived peptides as measured by IFN-γ ELISPOT. PBMC from melanoma patients were analyzed. Eight synthetic long Bcl-X(L)-derived peptides were examined. The average number of Bcl-X(L)-specific spots (after subtraction of background activity) was calculated per 10^5^ PBMC for each patient (*black circles*).

Hence, numerous long BCL-X(L) derived peptide epitopes exist, which may be used in anti-cancer immunotherapy. Thus, when validated, especially BclX5, BclX6 and BclX8 could be useful targets. Figure 4 gives an overview of the responses in all eight melanoma patients after subtraction of IFN-γ backgrounds.

## 4. Discussion

Most human cancers have defects in apoptosis signaling, which renders the cells unresponsive to stimuli that would otherwise inflict cell death. Apoptosis is strictly regulated in cells by a refined balance between pro-apoptotic and anti-apoptotic factors promoting and preventing apoptosis respectively. Many defects in malignant cells are caused by an up regulated expression of anti-apoptotic proteins. One such protein from the Bcl-2 family is Bcl-X(L). Increased expression of Bcl-X(L) has been described in various different malignancies including cancers of hematological origin such as acute myeloid leukemia and multiple myeloma but also in solid cancers like bladder cancer, breast cancer, pancreatic cancer and melanoma [10]. Bcl-X(L) has been directly linked to resistance to conventional forms of therapies and poor prognosis [10] .The functional inhibition of Bcl-X(L) restores the apoptotic process and renders neoplastic cells sensitive to chemical and radiation therapies, whereas manipulation of cancer cell lines to express high levels of Bcl-X(L) results in a multi-drug resistance phenotype. Thus, the attractiveness of targeting Bcl-X(L) in vaccination is based on the fact that down regulation or loss of expression of this protein as some form of immune escape would impair sustained tumor growth. The combination of immunotherapy targeting Bcl-X(L) with conventional chemotherapy appears to be particularly appealing since expression of this protein is correlated with drug resistance [11,12]. Previously, using the ELISPOT assays we demonstrated that cancer patients host spontaneous HLA class I-restricted T-cell responses specifically against Bcl-X(L)-derived peptides [7]. In the present study, we show that longer BCL-X(L) derived peptides likewise elicited responses in melanoma patients. Furthermore, these were frequent responses, since in all patients responses against several peptides could be detected. The use of autologous DC as antigen presenting cells in the ELISPOT seems to generate some background in some assays. This is probably due to non-specific activation of T cells by the *in vitro* generated DC, e.g., due to the presentation of serum-derived antigens on the surface of DC. An alternative could be to perform ELISPOT without antigen presenting cells. However, this would probably be less sensitive due to the lack of antigen-processing because of the low number of DC among human PBMC. In contrast to short peptides the longer peptides used in the present study cannot bind directly to the surface—at least not in the context of HLA class I-restricted responses—but need to be taken up and processed by antigen presenting cells. Hence, when using long peptides with antigen presenting cells in the ELISPOT possible both CD4- and CD8-restricted responses are detected. In contrast ELISPOT assays performed with only long peptides without antigen presenting cells probably mainly detect CD4^+^ T-cell responses, since the binding groove of class II HLA-molecule has open ends and, consequently, can bind longer peptides directly compared to class I HLA-molecules. Furthermore, the expression of class II HLA-molecules are more restricted than class I HLA-molecules, which are expressed by virtually all cells.

Hence, numerous long BCL-X(L) derived peptide epitopes exist, which may be used in anti-cancer immune therapy. When validated, the long peptides BclX5, BclX6 and BclX8 described here may be suitable candidates for future vaccine trials as they elicit responses. The IFN-γ ELISPOT assay is one of the most useful techniques for immunological monitoring of T-cell responses and has gained increased application as a measure of specific T cell activation. However, other cytokines could be included to further characterize the nature of the immune response, e.g., to identify poly-functional T cells pro-inflammatory cytokines like TNF-α and IL-2 would be informative. IL-17 has been of great interest recently owing to the discovery that the production of IL-17 characterizes subset of CD4^+^ T-helper cells (Th17 cells). Furthermore, IFN-γ secretion is not limited to only cytolytic cells (e.g., CD4^+^ T-helper cells release IFN-γ). GranzymeB (GrB) is a key mediator of target cell death via the granule-mediated pathway and the GrB ELISPOT assay was recently demonstrated to provide an estimation of cytotoxic effector cell frequency [13]. Additionally, unlike the IFN-γ ELISPOT assay, the GrB ELISPOT directly measures the release of a cytotolytic protein.

The long Bcl-X(L)-derived peptides that were subjects to immune responses were all located towards the C-terminal of the inserted region of the protein. Interestingly, the previously described HLA-A2-restricted Bcl-X(L) epitopes were likewise located in this part of the Bcl-X(L) sequence [7]. Only three of the patients in this study were HLA-A2 positive, which taken together suggest that the C-terminal part of the inserted region of Bcl-X(L) is particular immunogenic. In this respect, it has been described previously that T-cell epitopes often cluster together at specific immunogenic regions.

It has been repeatedly suggested that when used in the clinical setting, e.g., for anti-cancer vaccinations, longer peptides may elicit stronger response compared to 8–10 amino acids minimal epitopes. The longer peptides may specifically target professional antigen presenting cells, which are capable of the up taking and processing into HLA of larger peptide antigens. Hence, vaccination with short peptides is far from optimal because it can lead to immunological tolerance of the immunizing antigens, rather than immunity [14]. It was reported that an increase in the length of the peptide used for vaccination strongly affects the magnitude of the induced CD8^+^ T-cell response. In head-to-head comparisons, vaccination with long peptide (20–45 amino acids in length) epitopes resulted in more robust and effective T cell responses than vaccination with the minimal peptide epitopes [15]. Long peptides may contain both CD8 and CD4 T cell epitopes. CD4^+^ T helper cells have been shown to support tumor cell clearance through various mechanisms. T-helper cells support CTL responses in both the priming and the effector phase as well as in maintaining the CTL response. T-helper cells secrete cytokines essential for CD8^+^ T-cell proliferation and clonal expansion, e.g., IL-2. Th1 cells also secrete IFN-γ that result in MHC class I up regulation on tumor cells thereby further enhancing CTL recognition [16]. In addition, T-helper cells are able to exert some of their anti-tumor effects through the modulation of components of the innate immune system. The IFN-γ secreted by Th1 cells activates macrophages to produce nitric oxide and super-oxide both of which are important mediators of tumor cell killing [17]. 

In conclusion, we have identified several novel immunogenic epitopes from the tumor associated antigen Bcl-X(L). Furthermore, the ELISPOT assay offers an attractive and sensitive method for the characterization of spontaneous immune reactivity against long peptides. This might be useful for the identification of novel T-antigens and the characterization of highly immunogenic regions in such antigens. Finally, such long peptides might be more suitable for clinical applications than minimal epitopes.
